# A child with green urine after a diagnostic enema: Answers

**DOI:** 10.1007/s00467-021-05035-6

**Published:** 2021-03-17

**Authors:** Luisa Cortellazzo Wiel, Giulia Gortani, Davide Zanon, Matteo Bramuzzo, Marco Pennesi, Egidio Barbi

**Affiliations:** 1 University of Trieste, Trieste, Italy; 2 Institute for Maternal and Child Health – IRCCS “Burlo Garofolo”, Trieste, Italy

**Keywords:** Child, Green urine, Recurrent cystitis, Autoimmune gastritis, Cystoscopy, Enema, Methylene blue

## Answers

1. What is the differential diagnosis for a child with green urine?

The observation of green urine in this child raised further concerns about a recto-urethral fistula: however, this hypothesis was made unlikely by the lack of findings during cystoscopy, despite the irrigation of the rectum with a large amount of methylene blue. The differential diagnosis of green urine was therefore advocated. Congenital conditions potentially responsible for greenish discoloration of urine (namely, Hartnup disease, Blue diaper syndrome) were easily ruled out, based on the sudden occurrence of the phenomenon in adolescent age. Biliverdinuria and urinary tract infections were excluded by the normal urinalysis results. Among the different drugs potentially involved, propofol was taken into account: however, very low induction doses were used in this child, who separately had been sedated with propofol before, without displaying any similar findings. Finally, a dye-related urine discoloration was considered: based on previous reports, the occurrence of green urine in this child was attributed to the administration of methylene blue through a rectal enema. As expected, the urine discoloration eventually faded over few days. The history of recurrent cystitis was attributed to the severe constipation.

2. What diagnostic tests are useful to establish the diagnosis?

The diagnosis of dye-related urine discoloration is clinical and does not require any additional test. Urinalysis can assist the exclusion of urinary tract infections, potentially responsible for urine of greenish hue. The awareness of the benign nature of this condition prevented this child from undergoing further unnecessary invasive investigations.

3. How would you manage this patient?

Dye-related urine discoloration is self-limiting and does not require any additional treatment. The greenish hue tends to fade within a few days: patients must be reassured about the benign nature of the phenomenon.

## Discussion

Abnormal urine discoloration is always alarming to the patient and intriguing to physicians. A bluish or greenish hue can arise from either endogenous or exogenous factors [1]. The former includes congenital conditions impairing amino acid absorption from the gastrointestinal tract (e.g., Hartnup disease, Blue diaper syndrome), biliverdinuria (resulting from obstructive jaundice or biliary leak), and urinary tract infections sustained by *Pseudomonas* (due to the formation of pyocyanin pigment [2]). Several drugs with phenolic green chromophore derivatives, conjugated in the liver and excreted by the kidney [2], can cause a dose-related, self-limiting urine greenish discoloration [3]: they include propofol, mostly after prolonged intravenous infusions [4, 5], cimetidine, and promethazine [6]; in addition, less frequently implicated, non-phenolic compounds are metoclopramide [7], amitriptyline [8], and indomethacin [9].

Dyes such as food coloring [10], indigo blue, and methylene blue can sometimes be responsible. Methylene blue is a water-soluble dye used to treat conditions such as methemoglobinemia and ifosfamide-induced encephalopathy [11] and as a diagnostic aid to assess the integrity and patency of either the visceral walls or the intragastric balloons in bariatric surgery [12]. Moreover, it is often contained in several traditional Chinese medications, due to its antimicrobial properties [13]. After kidney filtration, blue pigments combine with urochrome, the major contributor to urine’s normal yellow hue, discoloring urine green before elimination [1]. While the peak of excretion is between 2 and 6 h after administration, it can be detected in urine through chromatographic techniques until 24–48 h after administration. Methylene blue–related greenish urine discoloration has been reported after several modes of administration of the dye: these include both direct intravenous infusion [11] and systemic absorption after oral administration [14, 15], uterine filling during laparoscopic chromopertubation for infertility work-up [16], colon submucosal injections [17], irrigation of the bowel lumen [18], and manipulation of the paralytic ileum [19]. To date, only one case of urine discoloration after a methylene blue enema has been described in adults [20], and no similar reports exist in the pediatric literature.

Awareness of the benign and self-limiting nature of this condition can prevent children from undergoing unnecessary invasive investigations.

## Data Availability

Data sharing is not applicable to this article as no datasets were generated or analyzed during the current study.
